# Neutrophil extracellular traps: from antimicrobial innate immunity to the development of chemotherapy-induced peripheral neuropathy

**DOI:** 10.1016/j.ebiom.2023.104526

**Published:** 2023-03-16

**Authors:** Haruki Koike, Yohei Iguchi, Kentaro Sahashi, Masahisa Katsuno

**Affiliations:** a Department of Neurology, Nagoya University Graduate School of Medicine, Nagoya, Japan; b Department of Clinical Research Education, Nagoya University Graduate School of Medicine, Nagoya, Japan

Neutrophils play a pivotal role in the innate immune system. They migrate and accumulate at the site of infection and eliminate microbes by phagocytosis, reactive oxygen species (ROS) production, and cytoplasmic granule release.^1^ Additionally, Brinkmann et al., in 2004 reported that even long chromatin fibers of neutrophils extruded into the extracellular space, leading to neutrophil extracellular trap (NET) formation, as another antimicrobial activity.^2^ This phenomenon is morphologically characterized by loss of plasma membrane, decondensed chromatin, and indistinct nuclear envelope of neutrophils and is called as NETosis because it is an active cell death mechanism distinct from necrosis and apoptosis.^3^ Recent studies suggested that harmful aspects of NETosis exist although it was initially considered a beneficial host defense mechanism.^1^ For example, excessive NET formation resulting from NETosis damages vascular endothelial cells, forms thrombi, and impairs diabetic wound healing.^3^ NET dysregulation can lead to autoimmune diseases such as systemic lupus erythematosus, anti-neutrophil cytoplasmic antibody-associated vasculitis, and psoriasis.^1^^,^^4^ Furthermore, NETs promote tumor growth, contribute to metastasis, and interfere with the action of cytotoxic lymphocytes.^3^ Multiple functions of NETs, ranging from positive roles in innate immunity to negative roles in the development of various diseases, have attracted the attention of many researchers.

In a recent issue of *eBioMedicine*, Wang et al. investigated the mechanisms of chemotherapy-induced peripheral neuropathy (CIPN) by focusing on NETs.^5^ Mice receiving oxaliplatin showed NET accumulation in the dorsal root ganglia and limbs and microcirculation disturbance in limbs and sciatic nerves. Therapeutic attempts targeting NETs significantly improved peripheral microcirculation disturbance and oxaliplatin-induced mechanical hyperalgesia. Additionally, an examination using plasma from human participants revealed a significant increase in the NET markers (i.e., citrullinated histone H3) in patients receiving chemotherapy. These results provide important insights into not only the pathophysiology but also the management of CIPN. CIPN has long been known as one of the major adverse events in patients receiving chemotherapy. Among many chemotherapeutic agents that may induce neuropathy, oxaliplatin is known to cause chronic sensory neuropathy resulting from the damage of neurons in the dorsal root ganglia and acute cold-induced dysesthesia in the face and hands.^6^^,^^7^ From the clinical viewpoint, CIPN is important because it interferes with the continuation of chemotherapy for patients with cancer and significantly disturbs the quality of life of patients due to neuropathic pain even after treatment discontinuation.^6^ Additionally, CIPN caused by some chemotherapeutic agents, including oxaliplatin, may develop even after drug discontinuation.^6^ Therefore, the development of therapeutic agents based on the specific mechanisms of CIPN is in urgent need. However, the mechanisms of CIPN have not been fully elucidated. The damage of neurons in the dorsal root ganglia has been considered to result from platinum-DNA adduct formation in oxaliplatin-induced neuropathy.^7^ By contrast, the acute symptoms of cold-induced dysesthesia may be due to voltage-gated sodium channel alteration.^7^ This study elucidated another mechanism of oxaliplatin-induced neuropathy. The disturbed microcirculation and ischemic status caused by NETs may indicate the release of intracellular components from leukocytes in the bloodstream. Hence, recent electron microscopic studies of sural nerves from patients with eosinophilic granulomatosis with polyangiitis demonstrated that the occurrence of intravascular eosinophil ETosis, which is a phenomenon similar to NETosis, is closely associated with the formation of thrombus within the vascular lumen.^8^^,^^9^

The results of this study suggest that the control of aberrant NET formation can be a potential therapeutic target for CIPN. Establishing a novel therapeutic strategy provides a great impact on cancer management, considering the prevalence and clinical importance of CIPN. Further studies are needed to validate the hypothesis drawn from this study. However, several compounds, including anti-inflammatory/immunomodulatory agents, anti-thrombotic agents, nicotinamide adenine dinucleotide phosphate/ROS inhibitors, nucleases, probiotics, and vitamin D, have been evaluated as potential therapeutics against NETs because NETs are associated with various diseases.^10^ One of the putative potential agents is a stroke-homing peptide-guided DNase1, as introduced in this study. However, NETs play an important role in the innate immune system. Thus, more basic research and applied clinical trials are needed to evaluate the safety and efficacy of the therapeutic agents against NETs because anti-NET compound administration may have unexpected adverse events.

## Contributors

Haruki Koike wrote the first draft, and all authors critically evaluated the manuscript. All authors read and approved the manuscript.

## Declaration of interests

None declared.
